# Ligand-activated PPARδ inhibits angiotensin II-stimulated hypertrophy of vascular smooth muscle cells by targeting ROS

**DOI:** 10.1371/journal.pone.0210482

**Published:** 2019-01-08

**Authors:** Eun Sil Kang, Jung Seok Hwang, Won Jin Lee, Gyeong Hee Lee, Mi-Jung Choi, Kyung Shin Paek, Dae-Seog Lim, Han Geuk Seo

**Affiliations:** 1 College of Sang-Huh Life Sciences, Konkuk University, Gwangjin-gu, Seoul, Korea; 2 Department of Nursing, Semyung University, Jechon, Korea; 3 Department of Biotechnology, CHA University, Bundang-gu, Seongnam, Korea; Medical College of Georgia at Augusta University, UNITED STATES

## Abstract

We investigated the effect of peroxisome proliferator-activated receptor δ (PPARδ) on angiotensin II (Ang II)-triggered hypertrophy of vascular smooth muscle cells (VSMCs). Activation of PPARδ by GW501516, a specific ligand of PPARδ, significantly inhibited Ang II-stimulated protein synthesis in a concentration-dependent manner, as determined by [^3^H]-leucine incorporation. GW501516-activated PPARδ also suppressed Ang II-induced generation of reactive oxygen species (ROS) in VSMCs. Transfection of small interfering RNA (siRNA) against PPARδ significantly reversed the effects of GW501516 on [^3^H]-leucine incorporation and ROS generation, indicating that PPARδ is involved in these effects. By contrast, these GW501516-mediated actions were potentiated in VSMCs transfected with siRNA against NADPH oxidase (NOX) 1 or 4, suggesting that ligand-activated PPARδ elicits these effects by modulating NOX-mediated ROS generation. The phosphatidylinositol 3-kinase inhibitor LY294002 also inhibited Ang II-stimulated [^3^H]-leucine incorporation and ROS generation by preventing membrane translocation of Rac1. These observations suggest that PPARδ is an endogenous modulator of Ang II-triggered hypertrophy of VSMCs, and is thus a potential target to treat vascular diseases associated with hypertrophic changes of VSMCs.

## Introduction

Vascular hypertrophy is characterized by thickening of vessel walls, which is mainly due to an increase in the size of vascular smooth muscle cells (VSMCs). These cells play critical roles in maintaining vessel integrity and tissue perfusion upon changes in arterial pressure associated with chronic hypertension [1, 2]. Among vasoactive mediators, angiotensin II (Ang II) promotes vascular hypertrophy via a process mediated by reactive oxygen species (ROS) derived from NADPH oxidase (NOX) [3–5]. With regard to the association of NOX with vascular hypertrophy, previous studies of genetically modified animals showed that pathological vascular hypertrophy is attenuated by ablation of NOX1 or NOX2, but enhanced by overexpression of NOX1 or p22^phox^, an essential component of NOX [3–5]. Although NOX-derived ROS play a central role in vascular pathogenesis by modulating hypertrophy of VSMCs [3–5], the molecular targets that can impede vascular hypertrophy are unclear [6].

Several reports demonstrated that peroxisome proliferator-activated receptor δ (PPARδ) is a potential target in the treatment of vascular disorders [7]. This nuclear receptor is reported to elicit anti-inflammatory and anti-atherosclerotic effects in the vasculature by regulating the availabilities of inflammation suppressors and the expression of extracellular matrix and proinflammatory genes [8, 9]. PPARδ elicits anti-inflammatory effects in an animal model of Ang II-induced atherosclerosis [10]. Ligand-activated PPARδ also inhibits the proliferation of VSMCs stimulated with interleukin (IL)-1β and tumor necrosis factor (TNF)-α by upregulating IL-1 receptor antagonist and transforming growth factor (TGF)-β1, respectively [11, 12]. Furthermore, the PPARδ ligand GW501516 inhibits oxidized low-density lipoprotein-stimulated proliferation and migration of VSMCs by upregulating SIRT1 expression [13]. In addition to its anti-inflammatory and anti-atherogenic effects, ligand-activated PPARδ inhibits oxidized low-density lipoprotein-induced apoptosis of VSMCs by activating anti-apoptotic signaling cascades such as the TGF-β and focal adhesion kinase pathways [14]. Recent reports also showed that PPARδ protects cardiomyoblasts and endothelial cells against oxidative stress-triggered apoptosis [15, 16] and inhibits Ang II-triggered premature senescence of VSMCs by upregulating antioxidant genes including manganese superoxide dismutase, glutathione peroxidase, thioredoxin, and heme oxygenase-1 [17]. The antioxidant activity of PPARδ in VSMCs involves transcriptional down-regulation of hydrogen peroxide-induced thrombospondin-1 expression [18]. Given the beneficial effects of PPARδ in the vasculature [8–14, 17, 18], it is important to assess its therapeutic potential for hypertrophic vascular disorders [7].

A recent study demonstrated that activated PPARδ attenuates Ang II-triggered hypertension by targeting regulator of G-protein-coupled receptor signaling 5 [19]. Based on this observation and our previous findings concerning the protective effects of PPARδ in the vasculature, we hypothesized that ligand-activated PPARδ may affect Ang II-induced vascular hypertrophy, a critical phenotype in hypertension. Accordingly, we investigated the effects of the PPARδ ligand GW501516 on Ang II-stimulated protein synthesis in VSMCs. Here, we show that activation of PPARδ by GW501516 inhibits Ang II-triggered protein synthesis in VSMCs by modulating the generation of ROS via a process involving activation of NOX by translocated Rac. These findings indicate that PPARδ is a potential molecular target to prevent vascular hypertrophy.

## Materials and methods

### Materials

Ang II and LY294002 (2-[4-morpholinyl]-8-phenyl-4H-1-benzopyran-4-one) were purchased from Calbiochem (La Jolla, CA, USA). GW501516 was obtained from Enzo Life Sciences (Farmingdale, NY, USA). L-[4,5-^3^H(N)]-leucine (54.1 Ci/mmol) was purchased from PerkinElmer (Boston, MA, USA). *N*-Acetyl-_L_-cysteine (NAC) was purchased from Sigma Chemical Co. (St. Louis, MO, USA). Diphenyleneiodonium chloride (DPI) was obtained from Dojindo Molecular Technologies (Kamimashiki-gun, Kumamoto-Ken, Japan). Dulbecco’s modified Eagle’s medium (DMEM), fetal bovine serum (FBS), and WelFect-si transfection reagent were obtained from WelGENE Inc. (Gyeongsan-si, Gyeongsangbuk-do, Republic of Korea). Chloromethyl-2′,7′-dichlorofluorescein diacetate (CM-H_2_DCF-DA) was purchased from Molecular Probes (Eugene, OR, USA). Polyclonal antibodies specific for p-Akt and Akt were obtained from Cell Signaling (Beverly, MA, USA). Polyclonal anti-NOX1 antibody and monoclonal antibodies specific for Na+/K+ ATPase α1 and NOX4 were purchased from Abcam (Cambridge, MA, USA). Monoclonal anti-PPARδ antibody and polyclonal anti-Rac1 antibody were obtained from Santa Cruz Biotechnology (Santa Cruz, CA, USA). A polyclonal horseradish peroxidase-conjugated rabbit IgG were purchased from GeneTex (Irvine, CA, USA).

### Cell culture

Rat aortic VSMCs were isolated from free-floating explants of aortae and maintained in DMEM containing antibiotics and 10% heat-inactivated FBS as described previously [13]. Cells were used at passage 8–16 in all experiments.

### Gene silencing using small interfering RNAs (siRNAs)

VSMCs were transfected with nonspecific control small interfering RNA (siRNA; Ambion, Austin, TX, USA) and with siRNAs against rat PPARδ (Ambion), rat NOX1 (5′-agatctatttctactggat-3′; Bioneer, Daejeon, Republic of Korea), and rat NOX4 (5′-aacgaaggggttaaacacc-3′, Bioneer) in serum-free medium using WelFect-si transfection reagent. The effects of gene silencing were analyzed further.

### Analysis of protein synthesis

Protein synthesis was analyzed by [^3^H]-leucine incorporation as described previously [20]. Briefly, VSMCs were seeded into 24-well plates at a density of 1 × 10^4^ cells per well and then synchronized to quiescence by serum starvation for 48 h. Following pretreatment with the indicated reagents or transfection of the indicated siRNAs, cells were stimulated with 500 nM Ang II in DMEM containing 0.1% FBS for 24 h and pulsed with 1 μCi/mL [^3^H]-leucine for the final 8 h. After washing with ice-cold PBS, cells were incubated with 5% trichloroacetic acid for 30 min. Trichloroacetic acid-precipitated materials were solubilized in 0.5 mL of 0.5 M sodium hydroxide containing 0.5% sodium dodecyl sulfate. Radioactivity was detected using a liquid scintillation counter (Beckman LS 6500, Fullerton, CA, USA).

### Assay of intracellular ROS

Intracellular ROS levels were determined using the fluorescent probe CM-H_2_DCF-DA. Cells seeded into 35 mm glass bottom dishes (SPL Life Sciences, Seoul, Korea) were transfected with siRNAs or pretreated with the indicated reagents for the indicated durations. Following treatment with GW501516, cells were exposed to Ang II and incubated with 10 μM CM-H_2_DCF-DA for the final 30 min. Green fluorescence corresponding to intracellular ROS was detected using a 520 nm long-pass filter and an Olympus FV-1000 laser confocal fluorescence microscope (Tokyo, Japan).

### Preparation of membrane, cytoplasmic, and total cell extracts

A Mem-PER Plus Membrane Protein Extraction Kit (Pierce Biotechnology, Rockford, IL, USA) was used to fractionate membrane and cytoplasmic proteins from cultured cells. Briefly, cells treated with the indicated reagents for the indicated durations were collected by scraping and centrifuged at 300 × *g* for 5 min. Collected cells were washed once with Cell Wash Solution by centrifugation at 300 × *g* for 5 min. After carefully removing the supernatant, the cell pellet was resuspended in Permeabilization Buffer and vortexed briefly to obtain a homogeneous cell suspension. After incubation for 10 min at 4°C, permeabilized cells were centrifuged at 16,000 × *g* for 15 min to obtain the cytosolic fraction. The resulting pellet was resuspended in Solubilization Buffer by pipetting and incubated for 30 min at 4°C. Following centrifugation at 16,000 × *g* for 15 min, the supernatant corresponding to the solubilized membrane fraction was collected. To prepare total cell extracts, cells were washed with ice-cold PBS and lysed in PRO-PREP Protein Extraction Solution (iNtRON Biotechnology, Seoul, Korea) containing 5 mM tetrasodium pyrophosphate, 10 mM sodium fluoride, 10 mM β-glycerophosphatase, and 1 mM sodium orthovanadate for 90 min at -20°C. Cell lysates were centrifuged at 10,000 × *g* for 20 min, and the supernatants corresponding to total cell extracts were collected. The protein concentration was determined by the Bradford method using bovine serum albumin as a standard.

### Rac1 pull-down assay

Levels of GTP-bound Rac1 (Rac1-GTP) were determined using a Rac1 Activation Assay Kit (Upstate Biotechnology Inc., Temecula, CA, USA) as described previously [21]. Briefly, cells in 100 mm culture dishes were pretreated with LY294002 or GW501516 for 30 min or 24 h, respectively, and then exposed to Ang II for 30 min. Following washing with MLB [Mg^2+^ lysis/wash buffer; 50 mM MgCl_2_, 5% Igepal CA-630, 750 mM NaCl, 5 mM EDTA, 10% (v/v) glycerol, and 125 mM Hepes, pH 7.5], cell lysates were incubated with glutathione-agarose beads conjugated with the Rac-binding domain of PAK (p21-activated kinase 1) for 6 h at 4°C. Bead-bound proteins were washed and eluted with SDS sample buffer. GTP-bound Rac1 was analyzed by immunoblotting with a monoclonal anti-Rac1 antibody.

### Western blot analysis

Aliquots of cell lysates were fractionated by SDS-polyacrylamide gel electrophoresis and transferred to an Immobilon-P transfer membrane (Millipore Corporation, Billerica, MA, USA). Membranes were blocked with 5% non-fat milk prepared in Tris-buffered saline (TBS) containing 0.1% Tween-20, incubated with the indicated specific antibodies diluted in TBS containing 0.01% Tween-20 overnight at 4°C, and then labeled with a peroxidase-conjugated antibody diluted 1:5,000 for 2 h at room temperature. Following extensive washing with TBS containing 0.1% Tween-20, immuno-reactive bands were detected using WesternBright ECL (Advansta, Menlo Park, CA, USA).

### Statistical analysis

Means were compared using the Student’s t-test. All data are expressed as means ± SE.

## Results

### GW501516-activated PPARδ inhibits Ang II-induced incorporation of [^3^H]-leucine in VSMCs

**Fig 1:**
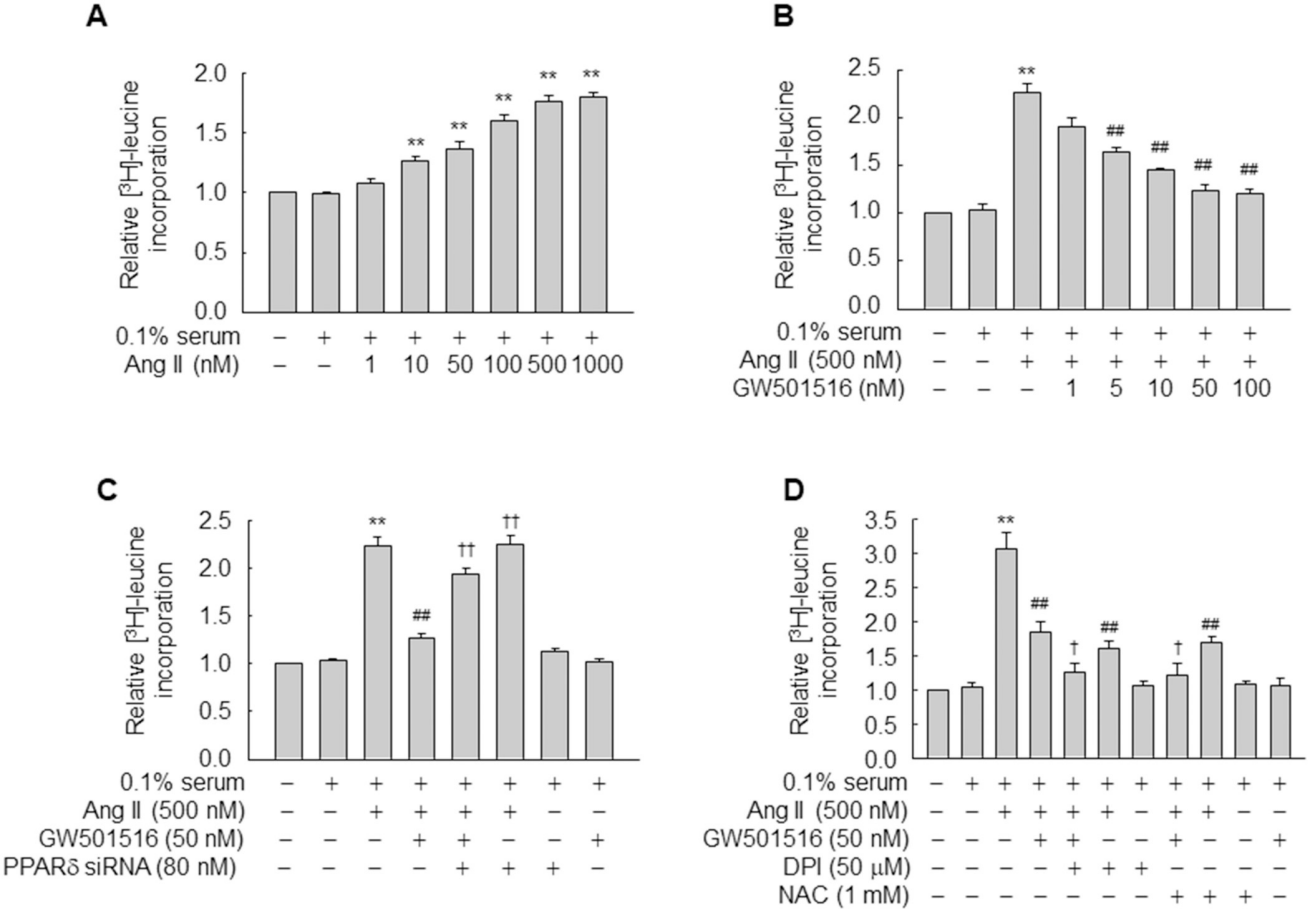
GW501516-activated PPARδ inhibits Ang II-induced incorporation of [^3^H]-leucine in VSMCs. (A) VSMCs were treated with increasing concentrations of Ang II for 24 h in DMEM containing 0.1% FBS. (B) VSMCs were pretreated with increasing concentrations of GW501516 for 24 h and then treated with Ang II for 24 h in DMEM containing 0.1% FBS. (C) VSMCs transfected with PPARδ-targeting siRNA for 48 h were pretreated with GW501516 for 24 h in DMEM containing 0.1% FBS and then exposed to Ang II for 24 h. (D) VSMCs pretreated with GW501516 (for 24 h), DPI (for 30 min), or NAC (for 30 min) were treated with Ang II for 24 h in DMEM containing 0.1% FBS and pulsed with 1 μCi/mL [^3^H]-leucine for the final 6 h. Data represent means ± SE (n = 4). **p<0.01 compared with the 0.1% serum-treated group; ^##^*p*<0.01 compared with the Ang II-treated group; and ^††^*p*<0.01, ^†^*p*<0.05 compared with the Ang II plus GW501516-treated group.

We examined whether GW501516, a specific ligand of PPARδ, affects Ang II-induced hypertrophy of VSMCs [2]. Exposure to Ang II significantly increased [^3^H]-leucine incorporation in VSMCs in a concentration-dependent manner (Fig 1A). GW501516 dose-dependently reduced this effect (Fig 1B), indicating that PPARδ inhibits Ang II-induced hypertrophy of VSMCs. This was confirmed by transfection of siRNA against PPARδ. The levels of PPARδ in VSMCs were dose-dependently reduced upon transfection with PPARδ siRNA, whereas control siRNA, consisting of a pool of nonspecific sequences, had no effect on the expression of PPARδ (S1 Fig). siRNA-mediated down-regulation of PPARδ almost completely abolished the inhibitory effect of GW501516 on [^3^H]-leucine incorporation (Fig 1C). Furthermore, pretreatment with DPI, an inhibitor of the flavin-containing enzyme including NOS [22], or NAC, a thiol antioxidant, significantly reduced incorporation of [^3^H]-leucine (Fig 1D), suggesting that NOX and ROS mediate Ang II-induced [^3^H]-leucine incorporation in VSMCs. These results indicate that PPARδ suppresses [^3^H]-leucine incorporation in VSMCs in a ROS-dependent manner.

### GW501516-activated PPARδ attenuates Ang II-induced generation of ROS in VSMCs

**Fig 2:**
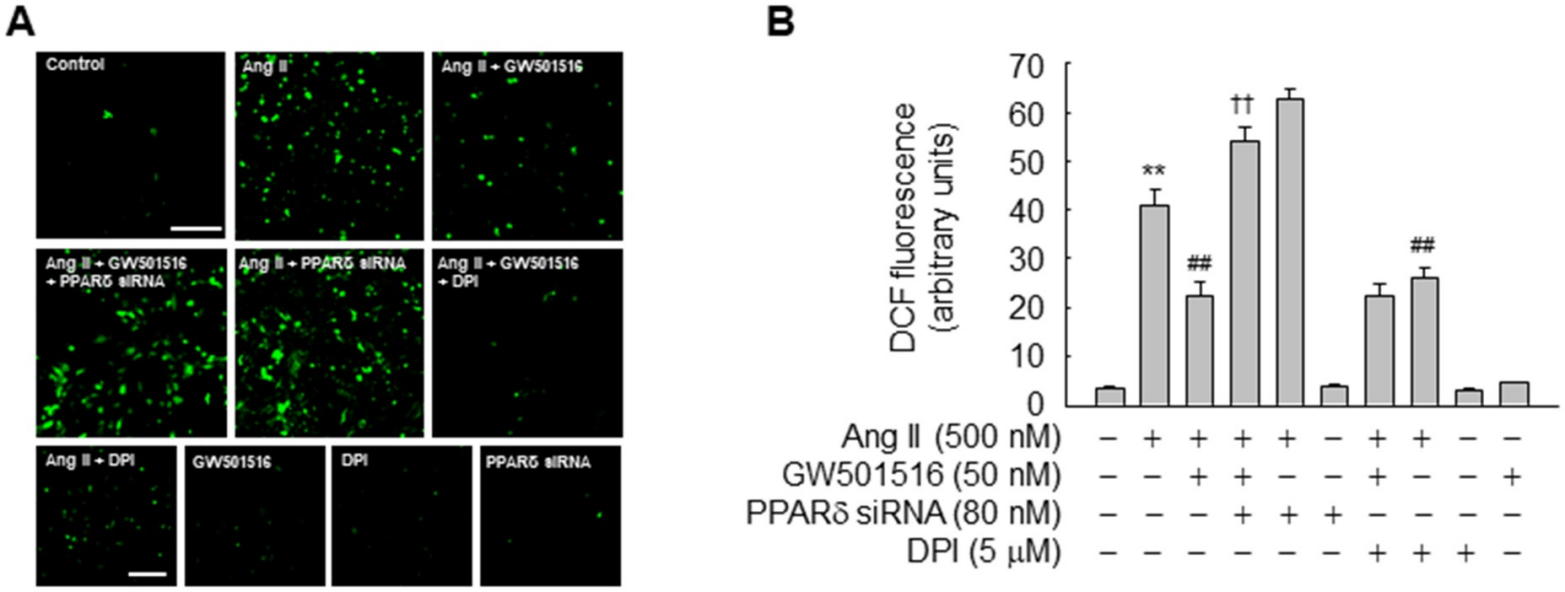
GW501516-activated PPARδ suppresses Ang II-induced generation of ROS in VSMCs. VSMCs transfected with PPARδ-targeting siRNA for 48 h or pretreated with DPI for 30 min were incubated in the presence or absence of GW501516 for 24 h and then treated with Ang II for 24 h. Intracellular ROS were detected by confocal laser fluorescence microscopy using the peroxide-sensitive dye CM-H_2_DCF-DA (10 μM, A), and the fluorescence intensity was quantified (B). Data represent means ± SE (n = 5). Bars, 200 μm. **p<0.01 compared with the untreated group; ^##^*p*<0.01 compared with the Ang II-treated group; and ^††^*p*<0.01 compared with the Ang II plus GW501516-treated group.

Ang II generates ROS via NOX in VSMCs [23]. Therefore, we evaluated the effects of GW501516 on ROS generation in Ang II-treated VSMCs. Ang II increased ROS production in VSMCs, and this effect was significantly inhibited by GW501516. This GW501516-mediated reduction in ROS generation was reversed in VSMCs transfected with PPARδ-targeting siRNA, indicating that PPARδ is involved in the effect of GW501516 on ROS generation. Furthermore, pretreatment with DPI reduced Ang II-induced ROS production similar to GW501516 (Fig 2A and 2B), suggesting that GW501516-activated PPARδ suppresses the production of NOX-derived ROS.

### NOX1 and NOX4 are involved in the effects of PPARδ on Ang II-induced [^3^H]-leucine incorporation and ROS production

**Fig 3:**
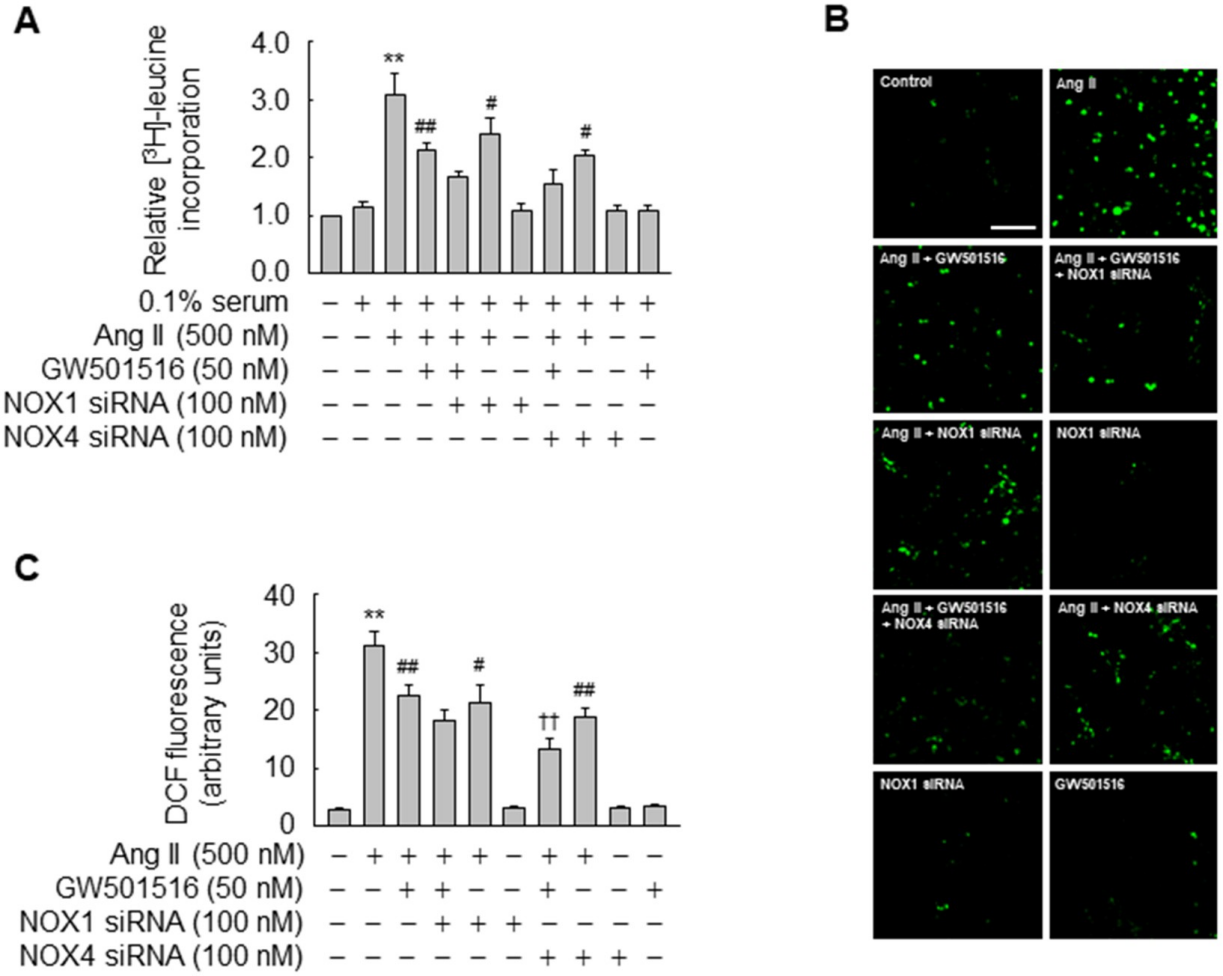
NOX is involved in the effects of GW501516 on VSMCs. (A) VSMCs transfected with siRNA against NOX1 or NOX4 for 48 h were pretreated with GW501516 for 24 h in DMEM containing 0.1% FBS. Thereafter, cells were treated with Ang II for 24 h and pulsed with 1 μCi/mL [^3^H]-leucine for the final 6 h. (B) VSMCs transfected with siRNA against NOX1 or NOX4 for 48 h were incubated in the presence or absence of GW501516 for 24 h and then exposed to Ang II for 24 h. Intracellular ROS were detected by confocal laser fluorescence microscopy using the peroxide-sensitive dye CM-H_2_DCF-DA (10 μM, B), and the fluorescence intensity was quantified (C). Data represent means ± SE (n = 5). Bars, 200 μm. **p<0.01 compared with the untreated group; ^##^*p*<0.01, ^#^*p*<0.05 compared with the Ang II-treated group; and ^††^*p*<0.01 compared with the Ang II plus GW501516-treated group.

We next investigated whether NOX is directly involved in GW501516-mediated suppression of Ang II-induced [^3^H]-leucine incorporation and ROS generation. VSMCs were stimulated with Ang II in the presence of GW501516 and/or siRNA against NOX1 or NOX4. In the presence of siRNA, the levels of NOX1 and NOX4 were markedly reduced, whereas expression was unaffected following transfection with control siRNA. The specificity of each siRNA was also cross checked on the expression of NOX1 and NOX4 (S2 Fig). [^3^H]-leucine incorporation and ROS generation were significantly increased in VSMCs exposed to Ang II, and these effects were diminished by pretreatment with GW501516 or transfection of siRNA against NOX1 or NOX4 (Fig 3). Ang II-induced [^3^H]-leucine incorporation tended to be reduced more by co-treatment with GW501516 and siRNA against NOX1 or NOX4 than by treatment with GW501516 alone (Fig 3A). However, significant effect on the ROS generation triggered by Ang II was observed in cells treated with GW501516 and siRNA against NOX 4, but not NOX 1 (Fig 3B and 3C).

### GW501516-activated PPARδ attenuates Ang II-induced [^3^H]-leucine incorporation and ROS generation by inhibiting phosphatidylinositol-3-kinase (PI3K)/Akt signaling

**Fig 4:**
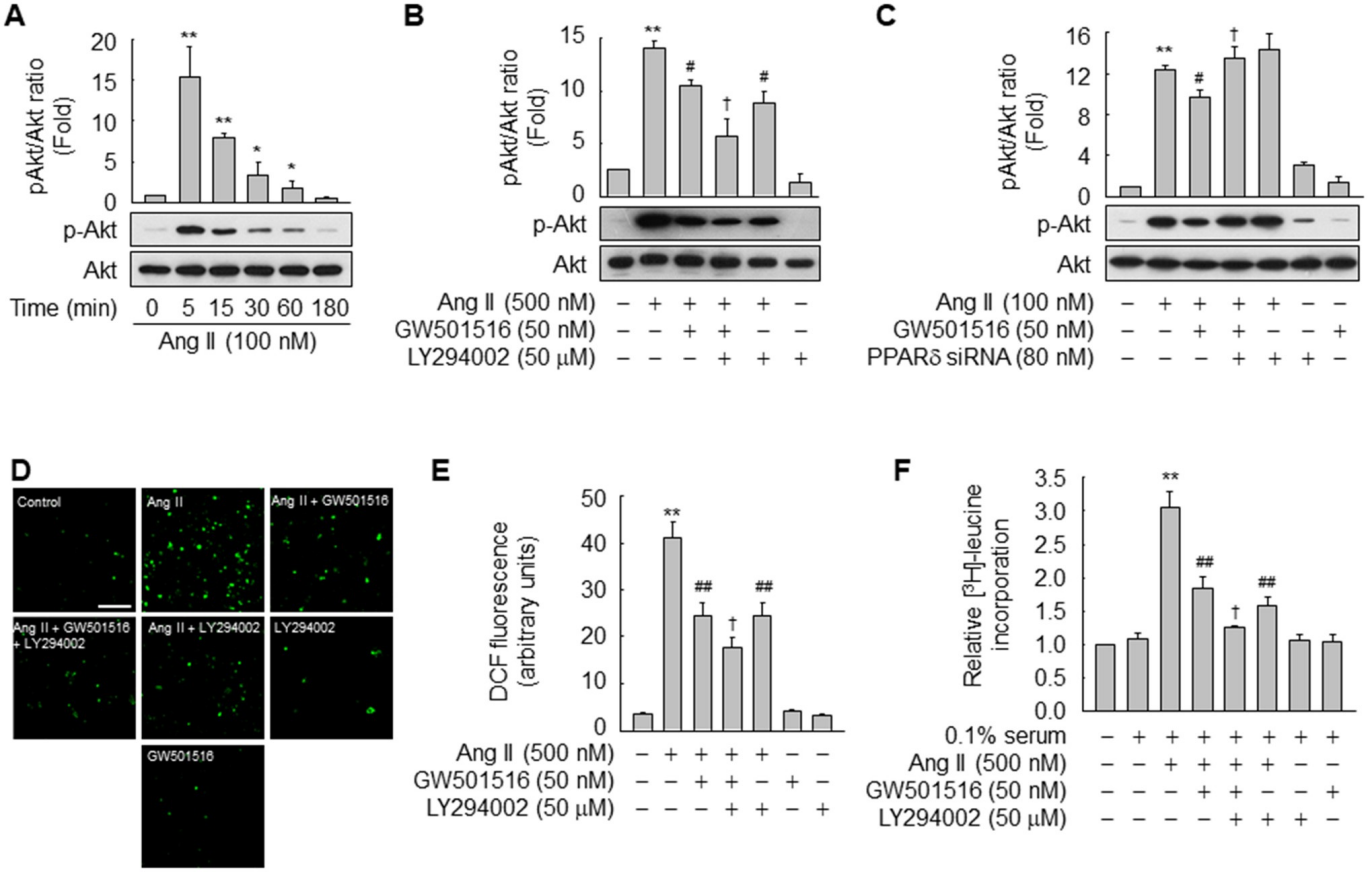
GW501516-activated PPARδ suppresses Ang II-induced ROS generation and [^3^H]-leucine incorporation in a PI3K/Akt-dependent manner. (A) VSMCs were treated with Ang II for the indicated durations. (B and C) VSMCs pretreated with LY294002 for 30 min (B) or transfected with PPARδ-targeting siRNA for 48 h (C) were incubated in the presence or absence of GW501516 for 24 h and then exposed to Ang II for 24 h. Levels of phosphorylated and total Akt were determined by western blotting. Representative blots from three independent experiments and densitometric measurements are shown. (D and E) VSMCs pretreated with LY294002 for 30 min were incubated in the presence or absence of GW501516 for 24 h and then exposed to Ang II for 24 h. Intracellular ROS were detected by confocal laser fluorescence microscopy using CM-H_2_DCF-DA (D), and the fluorescence intensity was quantified (E). Bars, 200 μm. (F) VSMCs pretreated with LY294002 for 30 min were incubated in the presence or absence of GW501516 for 24 h in DMEM containing 0.1% FBS. Thereafter, cells were exposed to Ang II for 24 h and pulsed with 1 μCi/mL [^3^H]-leucine for the final 6 h. Data represent means ± SE (n = 3 or 4). **p<0.01, *p<0.05 compared with the untreated group; ^##^*p*<0.01, ^#^*p*<0.05 compared with the Ang II-treated group; and ^†^*p*<0.05 compared with the Ang II plus GW501516-treated group.

The phosphatidylinositol-3-kinase (PI3K)/Akt signaling pathway is implicated in cellular oxidative stress and hypertrophy induced by Ang II [24]. Therefore, we investigated whether this pathway is involved in the inhibition of Ang II-induced [^3^H]-leucine incorporation and ROS generation by GW501516. Phosphorylation of Akt was induced upon exposure to Ang II, peaked at 5 min, and then decreased to the basal level over time (Fig 4A). Ang II-stimulated activation of Akt was markedly reduced by treatment with GW501516 or LY294002, and was reduced to a greater extent in the presence of both reagents (Fig 4B). On the other hand, siRNA-mediated down-regulation of PPARδ significantly reversed the reduction in Ang II-induced Akt phosphorylation by GW501516 (Fig 4C), suggesting that PPARδ modulates Ang II-activated PI3K/Akt signaling. To further evaluate the role of PI3K/Akt signaling in the actions of GW501516, we investigated the effects of PI3K/Akt blockade on Ang II-induced ROS generation and [^3^H]-leucine incorporation. Ang II exposure significantly increased the levels of ROS and [^3^H]-leucine incorporation in VSMCs, and these effects were significantly reduced by pretreatment with GW501516. LY294002 reduced the levels of ROS and [^3^H]-leucine incorporation to a similar extent as GW501516. The levels of ROS and [^3^H]-leucine incorporation were reduced more by co-treatment with GW501516 and LY294002 than by treatment with either reagent alone (Fig 4D–4F). These findings suggest that PPARδ and PI3K/Akt signaling regulate Ang II-induced protein synthesis and ROS production in VSMCs.

### GW501516-activated PPARδ inhibits Ang II-induced membrane translocation and activation of Rac1

**Fig 5:**
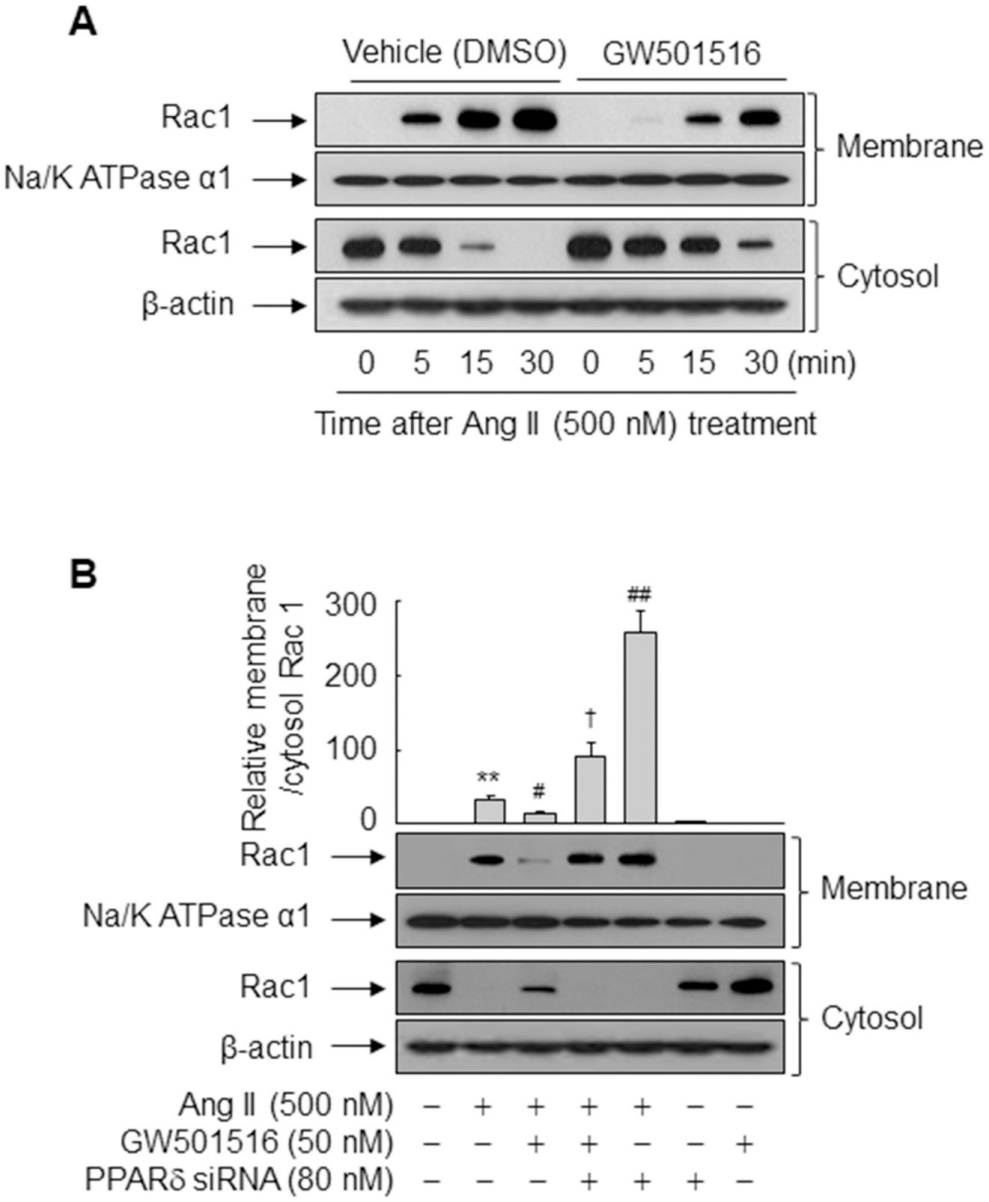
GW501516-activated PPARδ inhibits Ang II-induced membrane translocation of Rac1 in VSMCs. (A) VSMCs were treated with Ang II for the indicated durations in the presence of vehicle (DMSO) or GW501516. (B) VSMCs transfected with PPARδ-targeting siRNA for 48 h were pretreated with GW501516 for 24 h and then exposed to Ang II for 30 min. Western blot analysis was performed using antibodies for Rac1, Na^+^/K^+^ ATPase α1, and β-actin. Representative blots from three independent experiments are shown. The band intensities quantified by an image analyzer are plotted as a fold of membrane Rac1 to cytosolic Rac1 relative to the untreated group. Results represent means ± SE (n = 3). **p<0.01 compared with the untreated group; ^##^*p*<0.01, ^#^*p*<0.05 compared with the Ang II-treated group; and ^†^*p*<0.05 compared with the Ang II plus GW501516-treated group.

Translocation of Rac1 from the cytosol to membranes is essential for activation of NOX and subsequent ROS production [25]. Thus, we examined the effects of GW501516 on membrane translocation of Rac1 in Ang II-treated VSMCs with marker proteins Na^+^/K^+^ ATPase α1 and β-actin for membrane and cytosol fraction, respectively. Exposure to Ang II rapidly induced a shift in Rac1 from the cytosolic to the membrane fraction, and this effect was markedly reduced by GW501516 (Fig 5A). The GW501516-mediated reduction in Ang II-induced membrane translocation of Rac1 was significantly reversed in VSMCs transfected with siRNA against PPARδ (Fig 5B), indicating that PPARδ is involved in Ang II-Rac1 signaling.

**Fig 6:**
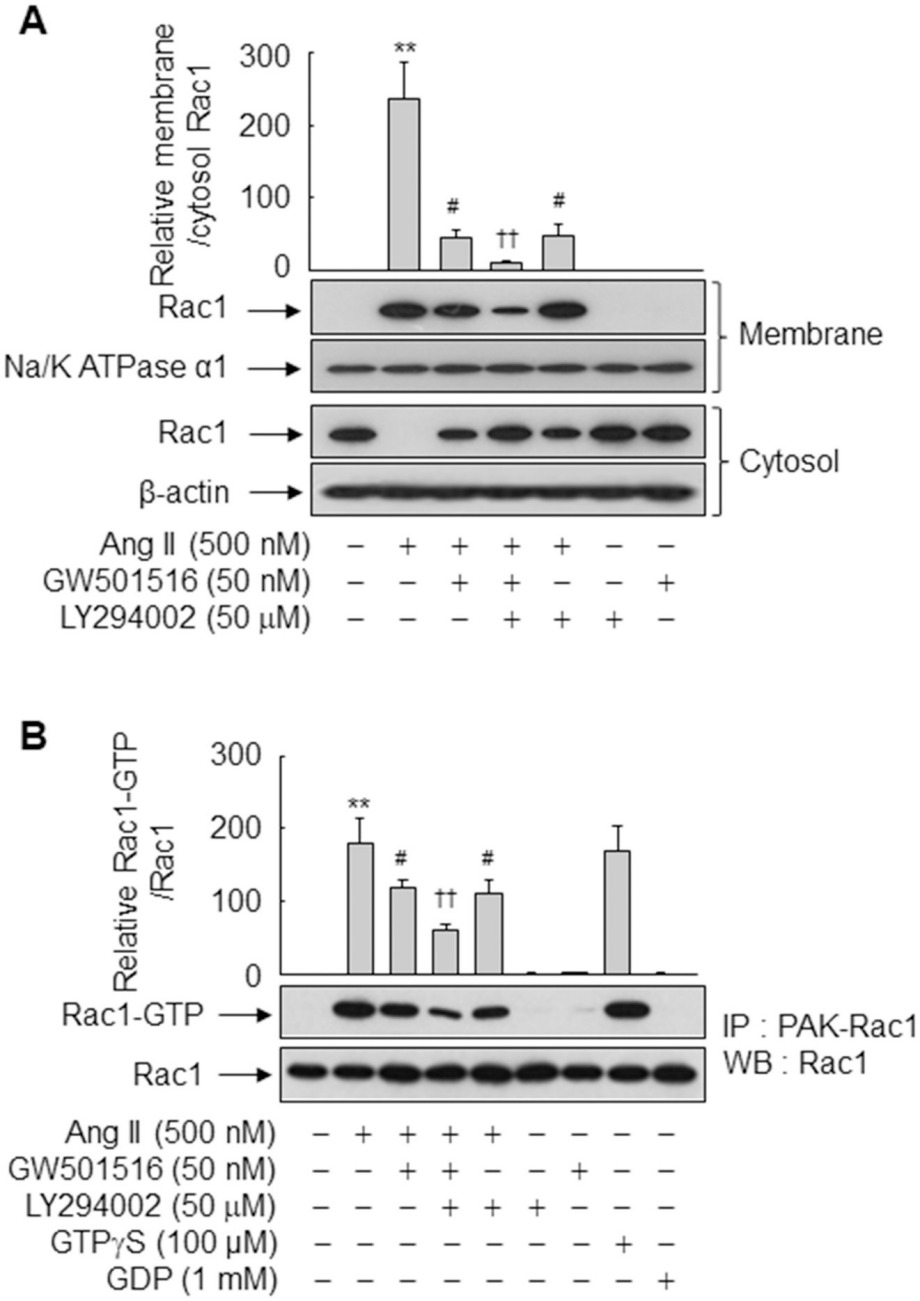
GW501516-activated PPARδ and PI3K inhibition prevent Ang II-induced activation of Rac1. (A) VSMCs pretreated with or without LY294002 for 30 min were incubated in the presence or absence of GW501516 for 24 h and subsequently exposed to Ang II for 30 min. Western blot analysis was performed using antibodies for Rac1, Na^+^/K^+^ ATPase α1, and β-actin. (B) VSMCs pretreated with or without LY294002 for 30 min were incubated in the presence or absence of GW501516 for 24 h, exposed to Ang II for 30 min, and harvested. The Rac1-GTP pull-down assay was performed using whole-cell lysates. Rac1-GTP levels were determined by immunoblotting. A fraction of the lysate was directly immunoblotted for normalization (Rac1). Representative blots from three independent experiments are shown. The band intensities quantified by an image analyzer are plotted as a fold of membrane Rac1 to cytosolic Rac1 relative to the untreated group. Results represent means ± SE (n = 3). **p<0.01 compared with the untreated group; ^#^*p*<0.05 compared with the Ang II-treated group; and ^††^*p*<0.01 compared with the Ang II plus GW501516-treated group.

The PI3K/Akt signaling cascade is involved in Ang II-induced ROS production [26]. Therefore, we investigated if blockade of PI3K/Akt signaling affects membrane translocation of Rac1 in VSMCs. Exposure to Ang II rapidly induced membrane translocation of Rac1, and this effect was significantly suppressed by GW501516 and LY294002. Ang II-induced membrane translocation of Rac1 was inhibited more by co-treatment with GW501516 and LY294002 than by treatment with either reagent alone (Fig 6A), suggesting that PI3K/Akt signaling is involved in Ang II-induced Rac1 signaling in VSMCs and that PPARδ plays a modulatory role.

To investigate the activity of Rac1 upon membrane translocation, activation of endogenous Rac1 was evaluated by pulling down Rac1-GTP. Ang II significantly activated Rac1, and this effect was suppressed by GW501516 and LY294002 (Fig 6B). The level of Rac1-GTP, an active form of Rac1, was reduced more by co-treatment with GW501516 and LY294002 than by treatment with either reagent alone. These data demonstrate that PPARδ and PI3K both affect Ang II-induced activation of Rac1 in VSMCs.

## Discussion

In addition to its genomic actions, a recent study demonstrated that the nuclear receptor PPARδ also has nongenomic actions, such as protein-protein interactions, and thereby elicits a wide spectrum of biological responses under pathophysiological conditions [27, 28]. Although PPARδ modulates the transcription of a diverse array of genes, little is known about which cellular signaling molecules it regulates. Here, we demonstrated that activation of PPARδ by GW501516 suppressed Ang II-induced [^3^H]-leucine incorporation in VSMCs by inhibiting the generation of ROS, which are key signaling molecules in hypertrophic changes of VSMCs [3–5]. PPARδ mediated this effect by blocking membrane translocation of Rac1, which is critical for the generation of ROS via NOX [29].

Our findings support the hypothesis that PPARδ has therapeutic potential for vascular disorders associated with hypertrophic changes of VSMCs. Ligand-activated PPARγ, a member of the PPAR family, inhibits Ang II-induced hypertrophy of VSMCs by down-regulating the type 1 Ang II receptor [30]. By contrast, inducible and conditional vascular-specific overexpression of PPARδ was reported to rapidly cause cardiac hypertrophy [31]. In addition, activation or knockout of PPARα, another member of the PPAR family, by application of an agonist or genetic manipulation improves cardiac hypertrophy induced by chronic pressure overload [32, 33]. Although the role of PPARs in vascular hypertrophy is controversial, our data clearly show that GW501516-activated PPARδ suppresses Ang II-induced hypertrophy of VSMCs.

The observation that GW501516-activated PPARδ suppressed Ang II-induced generation of ROS in VSMCs is consistent with our previous finding that PPARδ increases expression of antioxidant genes [17]. Similar effects are observed in VSMCs treated with NAC, indicating that ROS are key mediators of the effects of Ang II on vascular function [26]. The increases in blood pressure and endothelial dysfunction observed upon Ang II-induced hypertension are improved by activation of PPARα, which modulates NOX activity in vascular cells [34]. In addition, rosiglitazone, a specific ligand of PPARγ, protects the vasculature in hypercholesterolemic animals; specifically, it suppresses intracellular accumulation of ROS by regulating expression of PPARγ, gp91^phox^, and inducible nitric oxide synthase [35]. Although the molecular mechanisms by which PPARδ modulates the redox state in the vasculature are not well defined, our findings show that GW501516-activated PPARδ inhibits Ang II-triggered generation of ROS in VSMCs. ROS are thought to be a causal factor in vascular diseases [36]; therefore, it may be possible to ameliorate such diseases by blocking ROS generation in VSMCs using PPARδ.

This study also demonstrated that the PI3K/Akt survival pathway is involved in the effects of GW501516. Our previous studies showed that this pathway plays a pivotal role in PPARδ-mediated regulation of senescence induced by Ang II and UVB irradiation in VSMCs and keratinocytes, respectively [21, 26]. PPARδ is a member of the nuclear receptor superfamily and elicits diverse biological effects by regulating expression of its target genes [27]. In addition, PPARδ also has nongenomic actions, such as its interactions with other proteins, and we demonstrated that it protects the vasculature upon Ang II stimulation by inhibiting the PI3K/Akt pathway. By contrast, previous studies reported that PPARδ activates the PI3K/Akt pathway in several cell types including endothelial progenitor cells [37] and keratinocytes [38]. In fact, the level of phosphorylated Akt was increased in VSMCs pretreated with GW501516 for 30 min, whereas activation of Akt was attenuated by pretreatment with GW501516 for 24 h [26]. This suggests that the effects of GW501516 on the PI3K/Akt pathway vary according to the duration of treatment. The specificity and potency with which PPARδ affects its target genes are reported to differ according to the duration of PPARδ ligand exposure and the cell type [39].

Blockade of the membrane translocation of Rac1 by PPARδ is a key step in the modulation of Ang II-induced ROS generation by GW501516. NOX activated by Rac1 is the main source of ROS in vascular cells and is thought to function in a variety of pathophysiological disorders, such as vascular hypertrophy [40]. Multiple molecules, including Ang II, cytokines (e.g., TNF-α and IL-1β), and phosphodiesterase 2, induce intracellular accumulation of ROS via activation of Rac1-dependent NADPH [40–42]. However, the molecular targets that can disrupt this process have not been identified. This study provides evidence that GW501516-activated PPARδ prevents Ang II-induced generation of ROS in VSMCs by modulating membrane translocation of Rac1 and subsequent activation of NOX. Thus, PPARδ may attenuate hypertrophy by inhibiting membrane translocation of Rac1 and thereby preventing oxidative stress in the vasculature.

In conclusion, our observations indicate that PPARδ inhibits Ang II-induced hypertrophy of VSMCs by attenuating Rac1-mediated generation of ROS, which may be achieved, at least in part, through the PI3K/Akt signaling cascade. These data may enhance understanding of the molecular mechanisms underlying the anti-hypertrophic actions of PPARδ. In addition, they demonstrate that PPARδ is a potential therapeutic target for vascular disorders associated with cellular hypertrophy.

## Supporting information

S1 Fig Effects of siRNA on expression of PPARδ in VSMCs. Cells were transfected with indicated concentration of siRNA specific for PPARδ or control siRNA. Following incubation for 24 h, cells were harvested and an aliquot of total cell lysate was subjected to Western blot analysis. Expression of PPARδ was inhibited dose-dependently in the presence of PPARδ siRNA, but not control siRNA. M, molecular size markers.
(TIF)

S2 Fig Effects of siRNA on expression of NOX1 (A) and NOX4 (B) in VSMCs. Cells were transfected with indicated siRNA specific for NOX1, NOX4, or control siRNA. Following incubation for 24 h, cells were harvested and an aliquot of total cell lysate was subjected to Western blot analysis. Expression of NOX1 (A) and NOX4 (B) was inhibited in the presence of siRNA specific for NOX1 or NOX4, but not NOX4 or NOX1 siRNA, respectively. M, molecular size markers.
(TIF)
