# Supplementary figures and images for: Ligand-activated PPARδ inhibits angiotensin II-stimulated hypertrophy of vascular smooth muscle cells by targeting ROS
Source: PLoS One. 2019 Jan 8;14(1):e0210482. doi: 10.1371/journal.pone.0210482 (PMC6324793; doi:10.1371/journal.pone.0210482)

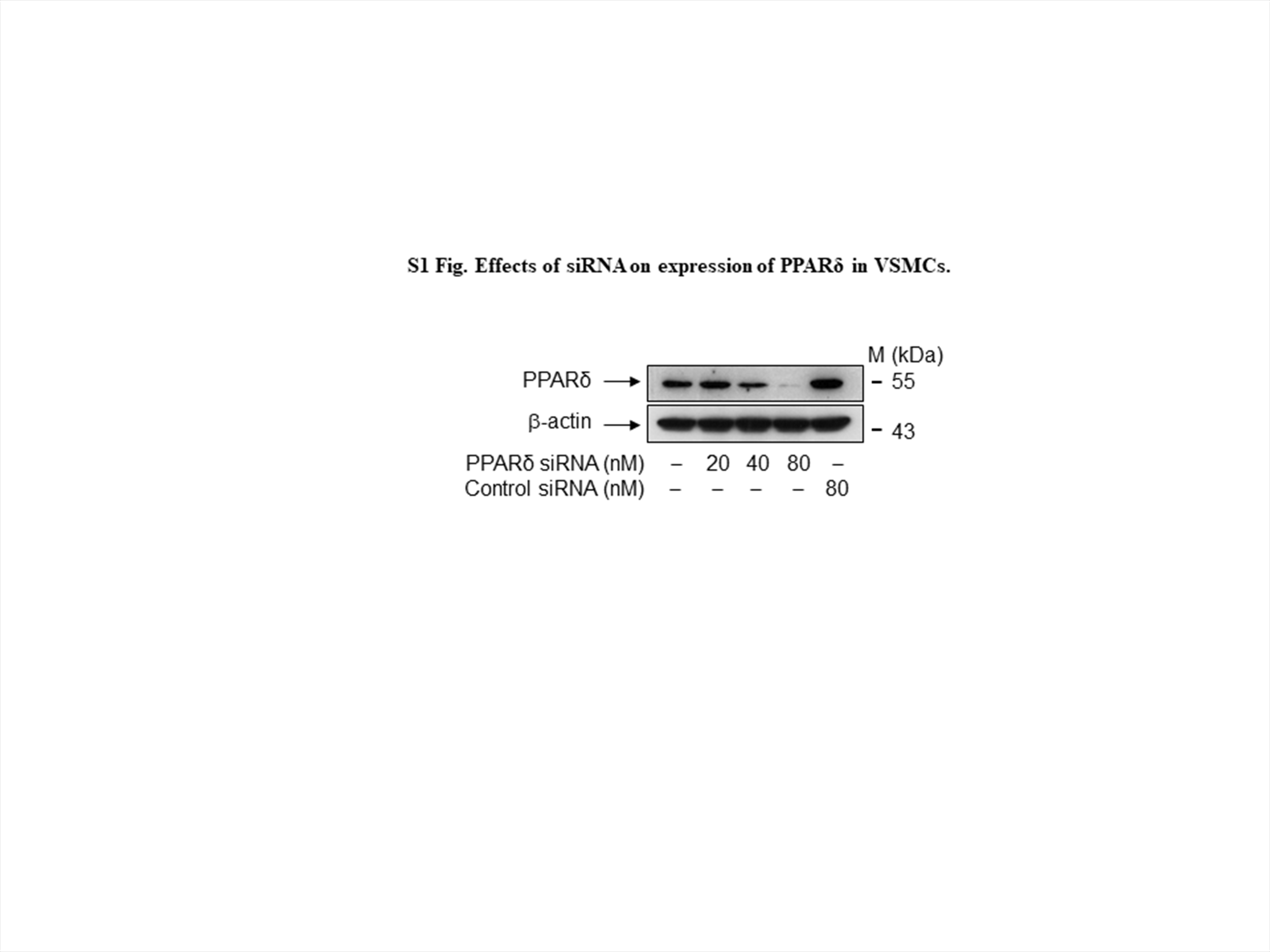

Supplement: S1 Fig — Cells were transfected with indicated concentration of siRNA specific for PPARδ or control siRNA. Following incubation for 24 h, cells were harvested and an aliquot of total cell lysate was subjected to Western blot analysis. Expression of PPARδ was inhibited dose-dependently in the presence of PPARδ siRNA, but not control siRNA. M, molecular size markers. (TIF) [file pone.0210482.s001.tif]

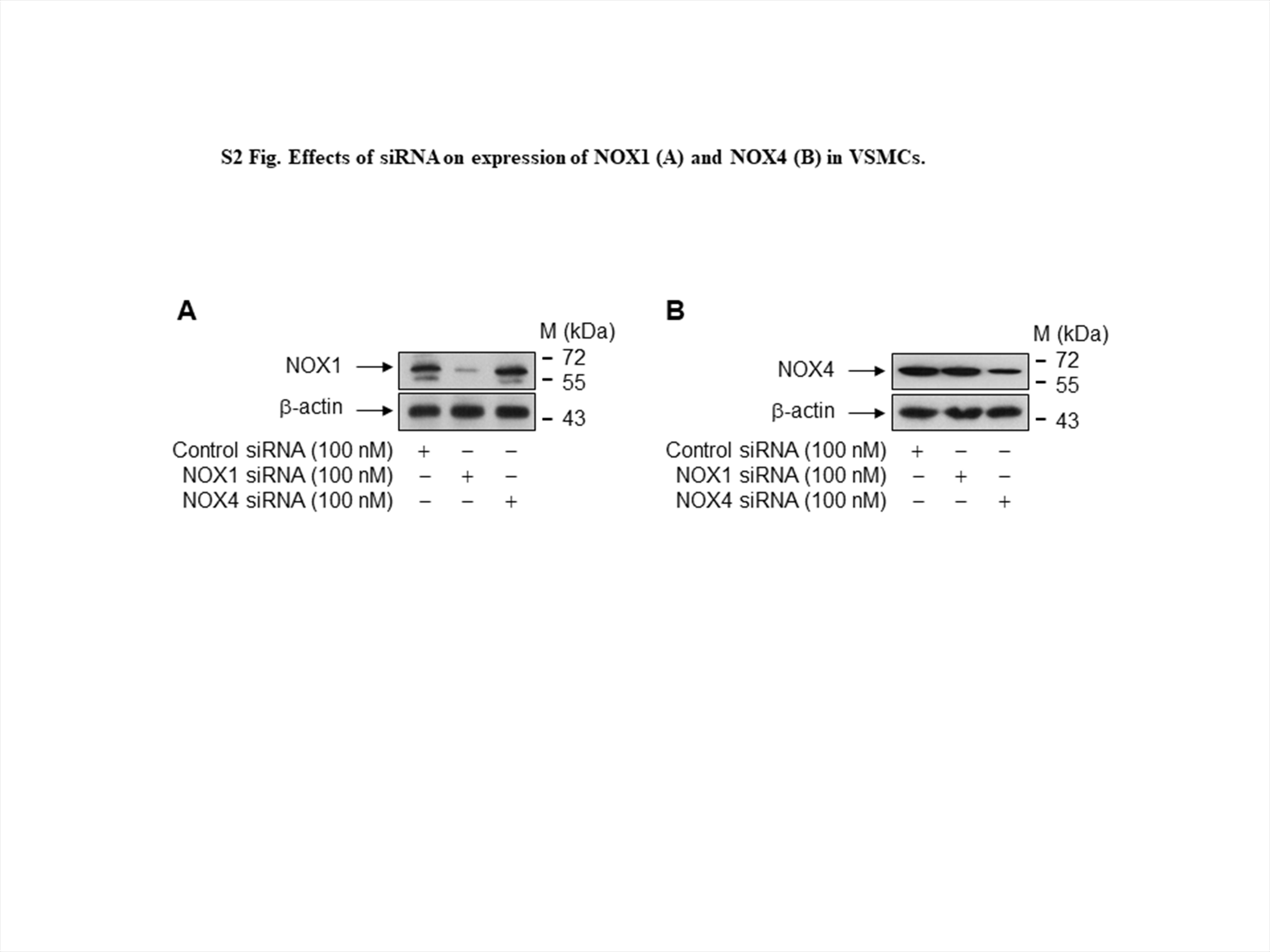

Supplement: S2 Fig — Cells were transfected with indicated siRNA specific for NOX1, NOX4, or control siRNA. Following incubation for 24 h, cells were harvested and an aliquot of total cell lysate was subjected to Western blot analysis. Expression of NOX1 (A) and NOX4 (B) was inhibited in the presence of siRNA specific for NOX1 or NOX4, but not NOX4 or NOX1 siRNA, respectively. M, molecular size markers. (TIF) [file pone.0210482.s002.tif]
